# Altered Metabolism and Inflammation Driven by Post-translational Modifications in Intervertebral Disc Degeneration

**DOI:** 10.34133/research.0350

**Published:** 2024-04-05

**Authors:** Dingchao Zhu, Huaizhen Liang, Zhi Du, Qian Liu, Gaocai Li, Weifeng Zhang, Di Wu, Xingyu Zhou, Yu Song, Cao Yang

**Affiliations:** ^1^ Department of Orthopaedics, Union Hospital, Tongji Medical College, Huazhong University of Science and Technology, Wuhan 430022, Hubei Province, China.; ^2^ College of Life Sciences, Wuhan University, Wuhan 430072, Hubei Province, China.

## Abstract

Intervertebral disc degeneration (IVDD) is a prevalent cause of low back pain and a leading contributor to disability. IVDD progression involves pathological shifts marked by low-grade inflammation, extracellular matrix remodeling, and metabolic disruptions characterized by heightened glycolytic pathways, mitochondrial dysfunction, and cellular senescence. Extensive posttranslational modifications of proteins within nucleus pulposus cells and chondrocytes play crucial roles in reshaping the intervertebral disc phenotype and orchestrating metabolism and inflammation in diverse contexts. This review focuses on the pivotal roles of phosphorylation, ubiquitination, acetylation, glycosylation, methylation, and lactylation in IVDD pathogenesis. It integrates the latest insights into various posttranslational modification-mediated metabolic and inflammatory signaling networks, laying the groundwork for targeted proteomics and metabolomics for IVDD treatment. The discussion also highlights unexplored territories, emphasizing the need for future research, particularly in understanding the role of lactylation in intervertebral disc health, an area currently shrouded in mystery.

## Introduction

Low back pain (LBP) is a degenerative disease that profoundly impacts the quality of life and places a substantial burden on society [1]. Various factors contribute to LBP, with intervertebral disc degeneration (IVDD) identified as the primary culprit [2]. Aging, prolonged overuse, and back injuries collectively induce degenerative changes in the intervertebral disc (IVD) [3]. These changes encompass the loss of collagen II and proteoglycan content, progressively disrupting normal spinal structure and function and ultimately leading to pain and disability [4]. The cartilage endplate (CEP)—a thin layer of hyaline cartilage situated between the vertebral body and IVD—undergoes structural alterations that impede the delivery of essential nutrients and oxygen to the IVD, emerging as another significant contributor to IVDD [5–6]. This cascade also results in an increase in metabolic intermediates for the biosynthesis of inflammatory and degradative proteins [7–8], activating key factors and inflammatory signaling pathways implicated in catabolic processes. Catabolic and hypoxic microenvironments further contribute to the degradation of the extracellular matrix (ECM) and the loss of homeostasis in the IVD microenvironment [9–10]. Moreover, mounting evidence suggests that IVDD involves low-grade inflammation and metabolic disturbances, prompting a shift in research focus toward metabolic features and changes in disease pathophysiology [11–12].

Nucleus pulposus cells (NPCs) within the degenerative IVD microenvironment and inflammatory conditions undergo pathological transformations marked by metabolic disorders and matrix remodeling [13]. These transformations are characterized by enhanced glycolysis pathways, mitochondrial dysfunction, and cellular senescence [14]. A shift toward glycolysis as the primary energy source results in lactate accumulation and a subsequent decrease in pH within the IVD, leading to tissue acidosis and ECM degradation [15]. Dysfunctional mitochondria generate excessive reactive oxygen species (ROS), which are the byproducts of oxidative phosphorylation [16]. ROS accumulation overwhelms cellular antioxidant defense mechanisms, resulting in oxidative stress and damage to cellular macromolecules, including lipids, proteins, and DNA, which activate inflammatory pathways and contribute to cellular senescence [17]. The accumulation of senescent cells within the disc tissue further exacerbates inflammation and matrix breakdown and impairs tissue repair processes associated with IVDD. In response to an increased nutrient supply, NPCs activate systems to boost synthesis output and promote energy storage and growth [18]. This metabolic response plays a crucial role in maintaining cellular homeostasis and supporting tissue integrity within the IVD [19]. Conversely, starvation triggers metabolic programs that inhibit biosynthetic pathways and activate catabolic energy-generating pathways [20]. These metabolic adaptations under nutrient deprivation conditions may lead to the breakdown of ECM components and compromise disc structural integrity, contributing to IVDD progression. Crucially, the activation of numerous hypoxia-responsive signaling pathways, essential for maintaining mitochondrial homeostasis and acid-base balance, underscores the characterization of IVDD by disturbances in intracellular metabolic regulation [21–22]. However, a detailed understanding of how metabolic alterations are induced during IVDD remains the subject of ongoing investigation.

**Fig. 1.**
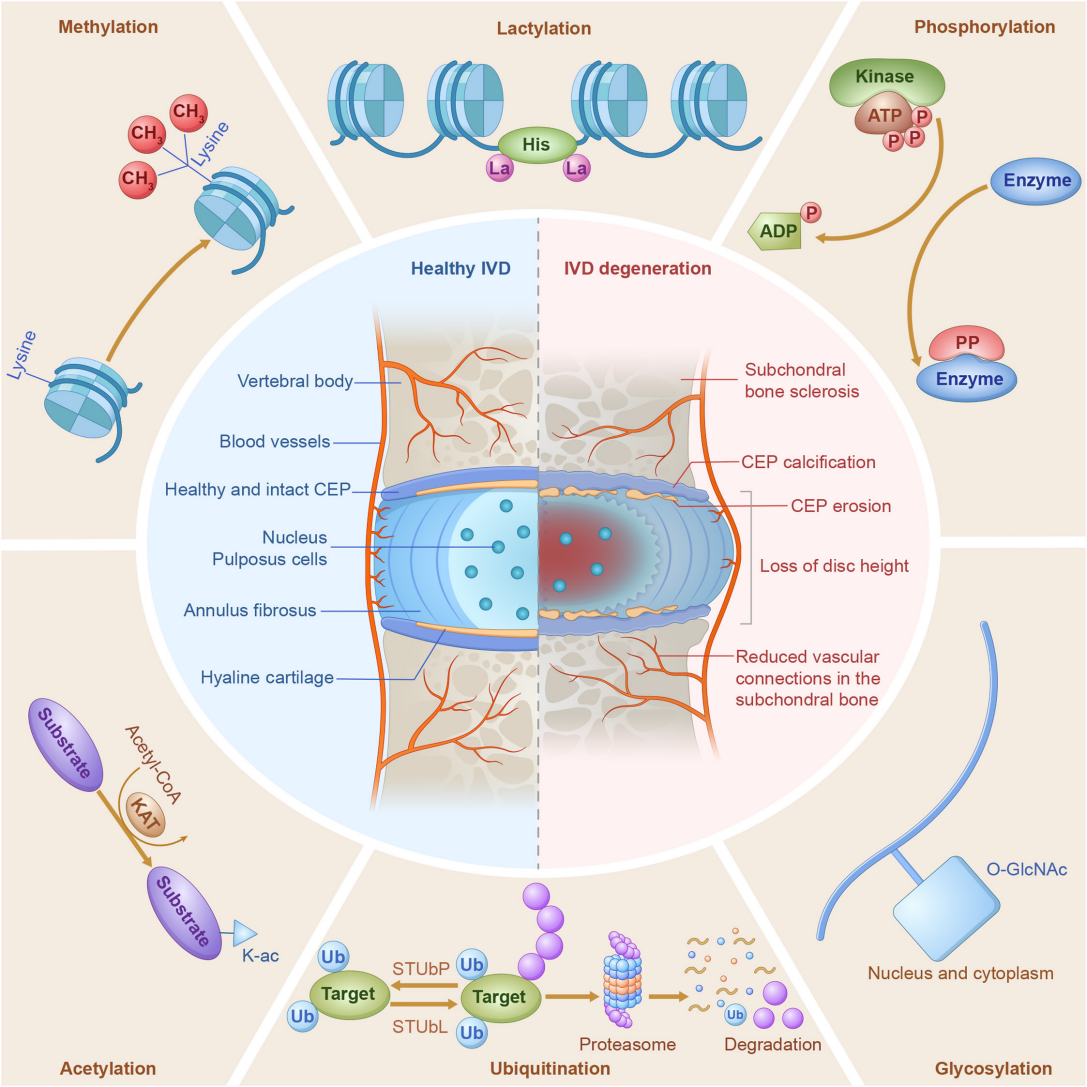
Representative PTMs in IVDD. IVDD is marked by depletion of the ECM, fibrous and dehydrated NP, severe structural alterations of collagen fibers in the AF, extensive cartilage damage, subchondral bone sclerosis, and a significant reduction in IVD height. This intricate process is influenced by various PTMs, including phosphorylation, ubiquitination, acetylation, glycosylation, methylation, and lactylation.

Metabolism can be regulated through changes in metabolic enzyme expression, substrate concentrations controlling reaction kinetics, or posttranslational modifications (PTMs) of proteins that facilitate these reactions [23–24]. These sequential events collectively govern the initiation, activation, and termination of metabolic reactions, determining the intensity of cell signaling pathways, cellular activities, tissue functions, and overall health and disease [25]. While the first two regulations establish key checkpoints reliant on meticulous regulatory mechanisms at the molecular level, regulation at the posttranslational level serves as a transducer of these metabolic signals and plays a pivotal role [26]. Traditional PTMs, such as phosphorylation and ubiquitination, have been shown to rapidly regulate protein stability and activity, ensuring the appropriate duration and extent of metabolism [27–28]. Unconventional PTMs, such as acetylation, glycosylation, and methylation, are increasingly implicated in the compartmentalization, transport, and physical interactions of key molecules, effectively regulating subcellular structure homeostasis and inflammatory responses [29–31]. This review focuses on the role of PTMs in IVDD (Fig 1). By delving into the mechanisms by which PTMs control metabolism and inflammatory responses in NPCs and chondrocytes, we consolidate the current state of knowledge in this field and provide a foundation for targeting proteomics and metabolomics to prevent IVDD. Furthermore, we identified priority areas for future research, particularly the potential role of lactylation in IVD, an area currently shrouded in mystery.

## Phosphorylation

### Phosphorylation regulates metabolic network

Phosphorylation, arguably the most prevalent and extensively studied PTM, is intricately linked to almost every cellular process [32]. The two primary mechanisms through which phosphorylation governs protein function involve the modulation of protein interactions and changes in protein conformation. This modification occurs through covalent binding of phosphate groups to serine, threonine, and tyrosine residues, catalyzed by protein kinases, and can be reversed by protein phosphatases [33]. Notably, robust communication between key kinases that regulate metabolism is a distinctive feature of the metabolic signaling network. For instance, AMP (adenosine monophosphate)-activated protein kinase (AMPK) and mammalian target of rapamycin (mTOR) jointly control ULK1 [34]. AMPK activation leads to a significant reduction in mTOR phosphorylation, subsequently activating ULK1 and initiating autophagosome assembly [35]. As a result, t-butyl hydroperoxide (TBHP)-induced autophagic influx is restored. Furthermore, AMPK-induced autophagy plays a crucial role in promoting ECM synthesis and inhibiting ECM degradation. This regulation occurs through the control of anabolic genes (e.g., collagen II and aggrecan) and catabolic genes [e.g., matrix metalloproteinase 13 (MMP13) and ADAMTS5], safeguarding against TBHP-induced ECM destruction [36]. Phosphorylated proteomics provides insights into the intricate network of signal processors. For instance, AMPK induces SIRT3 expression in human NPCs by phosphorylating PGC-1α [37]. Recent research has identified acetyl-CoA (coenzyme A) carboxylase (ACC) as a target of AMPK in lipopolysaccharide (LPS)-stimulated rat NPCs [38]. Through ACC phosphorylation, AMPK mitigates the generation of free radicals (ROS and nitric oxide) and production of proinflammatory cytokines (IL-1β and TNF-α). AMPK-mediated ACC activation also preserves aggrecan and collagen II content while inhibiting the expression of major ECM-degrading enzymes [39]. Independent phosphoproteomics studies have revealed that increased proteoglycans in the IVD inhibit the mTOR phosphatidylinositol 3-kinase (PI3K)/AKT signaling pathway, thereby promoting autophagy and reducing apoptosis in rat NPCs. Additionally, these studies have identified elevated BRD4 expression in NPCs of degenerative IVD. Silencing BRD4 activates the AMPK pathway, inhibits mTOR activity, promotes ULK1 phosphorylation, increases the LC3-ii/LC3-i ratio and Beclin-1 levels, and reduces P62 level in NPCs [40]. These findings suggest that autophagy mediated by mTOR activation can mitigate oxidative stress injury and impede IVDD progression. Recently, researchers found overloading-induced activation of ras homolog family member A-protein kinase N, which phosphorylates keratin 8 serine 43, subsequently impeding Golgi apparatus trafficking of the small guanosine triphosphatase RAB33B. This disruption inhibits autophagosome initiation and contributes to IVDD pathogenesis, suggesting a critical role of phosphorylation-regulated autophagy in IVDD [41].

### Metabolic stress-dependent phosphorylation

**Fig. 2.**
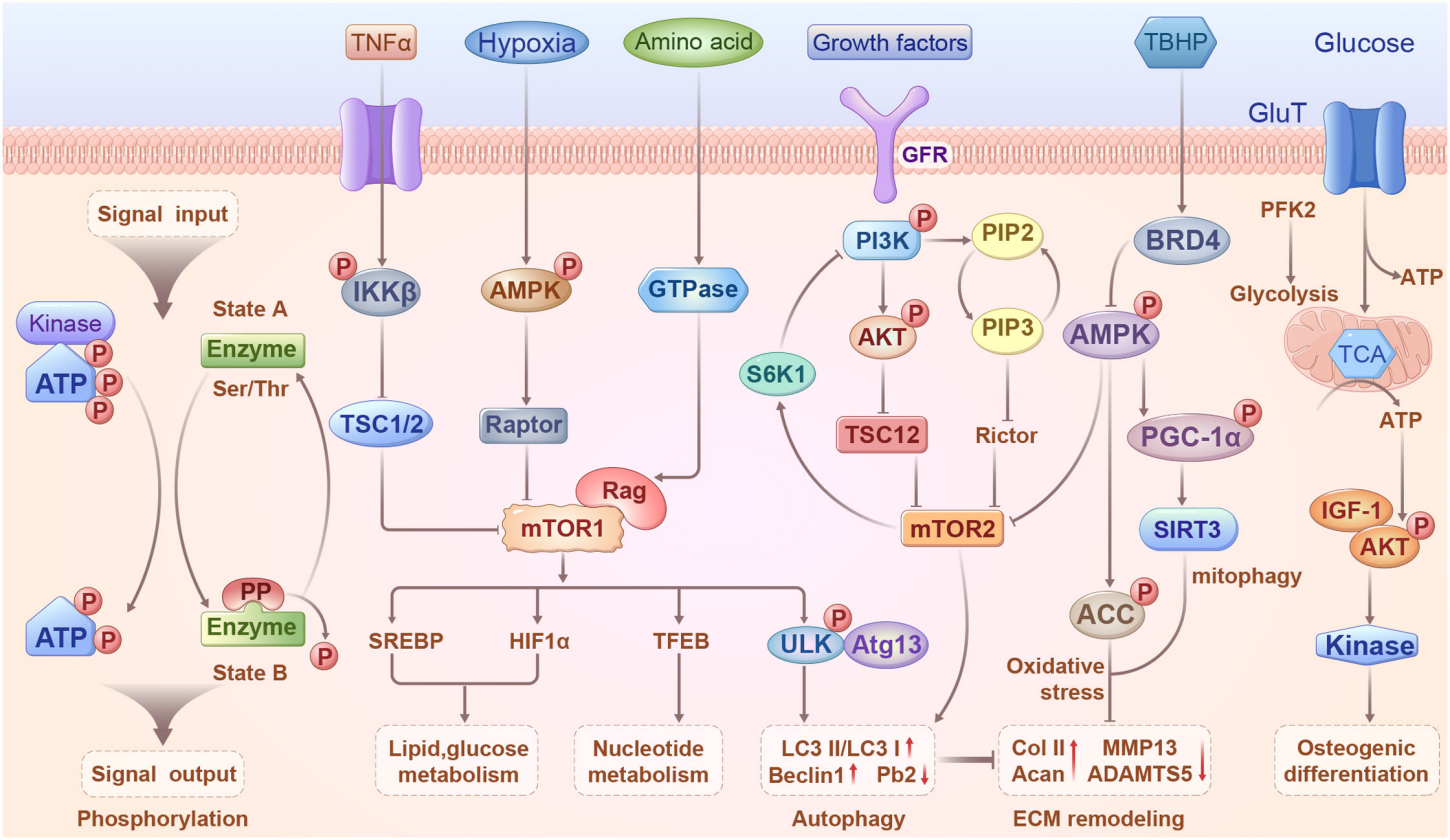
Interplay between phosphorylation and metabolism. Growth factors, hypoxia, stress, amino acids, and TNF-α activate mTORC1 phosphorylation, engaging in different signaling pathways that regulate lipid and glucose metabolism, nucleotide metabolism, and autophagy. Activation of mTORC2 is implicated in autophagy, cell survival, osteogenic differentiation, and aging. AMPK promotes apoptosis and senescence, inhibits cell proliferation, and retards cell cycle progression by inhibiting mTOR. Additionally, it inhibits oxidative stress, inflammation, and ECM destruction in IVDD through the activation of ACC. AMPK induces autophagy in IVD by regulating the mTOR-ULK1 pathway, promoting mitophagy, and inhibiting oxidative stress and inflammation in IVDD by activating PGC-1α and SIRT3. Furthermore, AMPK may shield IVDD from intrinsic apoptosis, senescence, and ECM destruction by maintaining mitochondrial homeostasis.

A burgeoning paradigm in cell signaling asserts that beyond merely regulating metabolism, signaling is reciprocally governed by metabolic processes [42]. Intracellular AMP levels increase in response to energy deprivation or metabolic stress stimuli. Elevated AMP binds to the γ-subunit of AMPK and induces a conformational change [43]. This alteration enables the phosphorylation of a threonine residue by a specific kinase on the catalytic α-subunit, ultimately activating AMPK [44]. NPCs dynamically modulate their metabolic rate to establish novel metabolic homeostasis by reconciling adenosine triphosphatase (ATP) demand and supply pathways, potentially regulated by the activation of the mTOR signaling pathway. Intriguingly, NPCs may enhance autophagic flux, eliminate intracellular ROS, and mitigate cellular damage by suppressing mTOR activity through continuous exposure to adverse factors [45]. Crucially, AMPK curtails mTORC1 activity by phosphorylating TSC2 and Raptor, key members of the mTORC1 pathway, thereby restraining protein and lipid synthesis and limiting cell growth [46–47]. In the context of obesity, excess substrate supplied to insulin-sensitive cells induces mitochondrial hyperactivity, leading to ATP overproduction and AMPK inhibition [48]. Concurrently, obesity often coincides with reduced secretion of the endogenous AMPK activator adiponectin, which contributes to IVDD [49]. Furthermore, mTORC1 can sense amino acids via the Rag complex, a regulator of the lysosomal surface [50–52]. AKT activity—a controller of cell growth and proliferation—is directly regulated by metabolism through an ATP-dependent allosteric switch [53]. Investigations have revealed that this ATP-dependent conformational change in AKT governs the pathway of inhibitory phosphatases, modulating kinase activity in response to cellular ATP levels [54–56]. Glycolysis also exerts a profound and direct influence on signaling. For instance, the phosphofructokinase 2 isoform positively regulates glycolytic flux and modulates insulin-like growth factor-1 (IGF-1) [57]. Meanwhile, IGF-1/AKT sustains anabolism and prevents endplate calcification by down-regulating carbonic anhydrase in degenerative CEP (Fig 2). In summary, phosphorylation serves as a docking site for intramolecular and intermolecular protein interactions. It can directly regulate enzyme activity by inducing changes in the protein conformation. Furthermore, phosphorylation governs the subcellular localization and turnover of its targets, influencing signaling in conjunction with other PTMs.

## Ubiquitination

### Ubiquitin: Inflammation regulator

Ubiquitin—a ubiquitous small regulatory protein in eukaryotes—exerts its influence by covalently attaching to substrates through the coordinated activities of the E1-E2-E3 enzyme ubiquitin thioester cascade [58]. This process involves the participation of E1 ubiquitin-activating enzyme, E2 ubiquitin-conjugating enzymes, and E3 ubiquitin-protein ligases [59–60]. Ubiquitin serves as a crucial regulator of substrate stability and signaling, playing a role in temporal, spatial, and specific contexts with significant implications for health and disease [61–63]. A20—identified as a dual-function ubiquitin regulatory enzyme—possesses ubiquitin E3 ligase and deubiquitinase capabilities and functions as a linear ubiquitin effector [64–65]. Mutations leading to impaired A20 protein function are associated with autoimmune and autoinflammatory phenotypes, given their role as inflammation suppressors. For instance, A20 plays a pivotal role in attenuating NLRP3 inflammasome-mediated apoptosis in NPCs by promoting mitophagy and stabilizing mitochondrial dynamics [66–67]. Targeting NLRP3 ubiquitination has emerged as a potential therapeutic approach for inhibiting NPC apoptosis. Platelet-rich derived exosomes facilitate NLRP3 autophagic degradation by enhancing NLRP3 ubiquitination, thereby reducing IL-1β and caspase-1 production and slowing IVDD progression [68]. Knockdown of ubiquitin-specific peptidase 14 (USP14) to induce NLRP3 ubiquitination inhibits pyroptosis in chondrocytes located in the annulus fibrosus (AF) [69]. X-linked inhibitor of apoptosis protein (XIAP)—an E3 ubiquitin-protein ligase—governs both the cell death pathway and immune signaling events dependent on nuclear factor κB (NF-κB) [70]. It facilitates NOD2 proinflammatory communication by promoting K63-linked RIPK2 polyubiquitination in the NOD2 communication complex, attracting LUBAC, and intensifying the generation of cytokines that are dependent on NF-κB and mitogen-activated protein kinase (MAPK). Reduced XIAP expression is directly correlated with excessive ECM apoptosis and an imbalance in the synthesis of catabolic factors [71]. SIAH1 targets and ubiquitinates XIAP, mediating senescence, apoptosis, increased inflammation, and decreased ECM synthesis in NPCs [72].

### Ubiquitination regulates oxidative stress

**Fig. 3.**
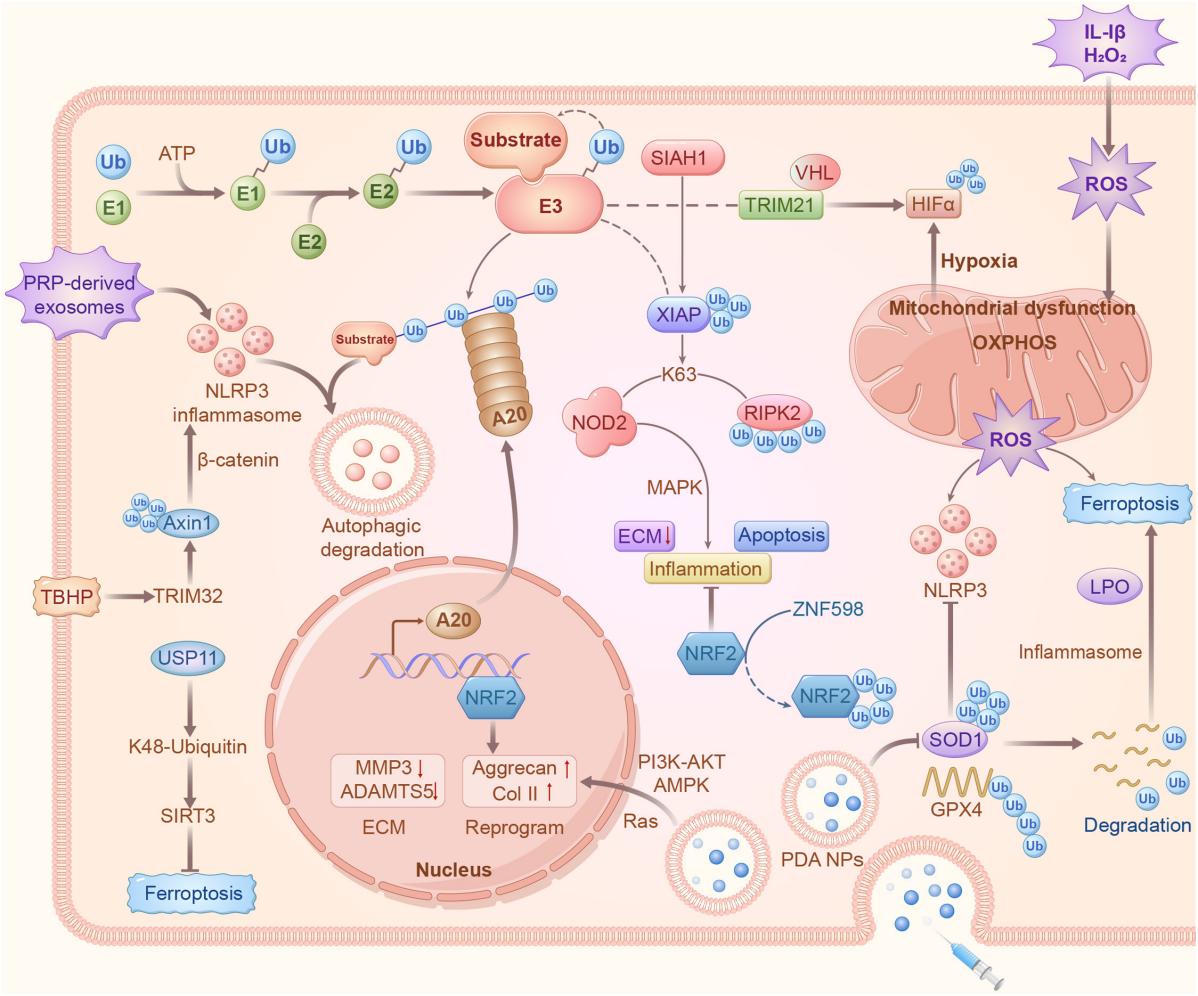
Ubiquitination regulation of inflammation and oxidative stress. The process involves successive enzyme interactions: ubiquitin activation and thioester formation by the E1-activating enzyme, ubiquitin trans-thiylation by the E2-conjugating enzyme, and ubiquitin attachment to the substrate assisted by E3 ligase. A20, a bifunctional ubiquitin regulatory enzyme, plays a role in inflammatory factor-induced apoptosis and pyroptosis in NPCs. The pivotal molecular event involves the direct interaction between USP11 and SIRT3, inhibiting oxidative stress-induced ferroptosis, improving IVDD, and alleviating the pain response. PDA NPs, functioning as a biological material, colocalize with GPX4 around the mitochondria to impede ubiquitin-mediated degradation, offering protective effects through the conversion and clearance of phospholipid hydroperoxides.

Maintaining a delicate balance between ROS and antioxidants is crucial for the normal function of IVD [73]. LPS induces ROS production and catabolism in rat endplate chondrocytes, triggering the secretion of inflammatory factors by macrophages and contributing to inflammation-related IVDD [74–75]. This process is dependent on ZNF598-mediated NRF2 ubiquitination [76]. Prussian blue nanoparticles enhance the mitochondrial structure and antioxidant capacity by stabilizing the ubiquitin-proteasome degradation of superoxide dismutase 1 (SOD1). This, in turn, increases mRNA and protein levels related to the oxidoreductase system, ultimately rescuing ROS-induced IVDD [77]. Conditional deletion of the E3 ubiquitin ligase von Hippel-Lindau tumor suppressor (VHL) in the CEP and AF of adult mice results in up-regulation of hypoxia-inducible factor-1α (HIF-1α) expression and age-dependent IVDD [78]. TRIM21 acts as a ubiquitin E3 ligase and drives oxidative stress-induced IVDD by promoting HIF-1α degradation [79]. Furthermore, TBHP induced the up-regulation of TRIM32 expression in rat NPCs, activating the β-catenin signaling pathway through the ubiquitination of Axin1, thereby regulating NPC apoptosis [80]. Lipid peroxidation (LPO) is a consequence of the interaction between ROS and lipids. Ferroptosis—a form of programmed cell death characterized by glutathione depletion and GPX4 inactivation—results in LPO accumulation [81]. Due to similar pathological processes, the interplay between oxidative stress and ferroptosis has been investigated in IVDD [82]. Mass spectrometry revealed that USP11 maintains stability by directly binding and deubiquitinating SIRT3, significantly improving oxidative stress-induced ferroptosis and alleviating IVDD [83]. Additionally, polydopamine (PDA) nanoparticles (NPs) colocalize with GPX4 around mitochondria, inhibiting ubiquitin-mediated degradation and down-regulating malondialdehyde and LPO production [84]. In this process, ROS, LPO, and ferroptosis represent direct responses to oxidative stress, whereas HIF-1α and mitochondrial homeostasis represent indirect responses to oxidative stress (Fig. 3). Ubiquitination regulates the effects of oxidative stress on NPCs and chondrocytes by modulating their metabolic state and cell survival mechanisms. The unique and time-dependent pattern of ubiquitination underscores its significance as a regulatory process that explains alterations in signaling and cellular milieu.

## Acetylation

### The multifunction of sirtuins

Acetylation is governed by two classes of enzymes: lysine acetyltransferases, which are responsible for transferring acetyl moieties to lysine residues, and lysine deacetylases, which catalyze acetyl group removal. Among these, sirtuins are a highly conserved class of nicotinamide adenine dinucleotide (NAD^+^)-dependent histone deacetylases (HDACs) that collectively regulate a diverse array of cellular functions [85–87]. SIRT1, a potent inhibitor of inflammation, deacetylates lysine residues on the p65 subunit of NF-κB, thereby inhibiting its transcriptional activity [88]. SIRT2 plays a crucial role in glucose homeostasis through deacetylation and activation of glucose-6-phosphatase dehydrogenase [89]. SIRT3, a vital mitochondrial deacetylase, plays a pivotal role in promoting fatty acid oxidation, Krebs cycle, and urea cycle [90]. Additionally, SIRT3 provides metabolic benefits and safeguards against metabolic dysregulation and oxidative stress associated with obesity [91]. SIRT4 controls glutamine breakdown [92], fatty acid oxidation [93], and lipid breakdown by deacetylating malonyl-CoA decarboxylase. SIRT5 selectively focuses on modifications other than lysine acetylation, including malonylation [94], succinylation [95], and glutarylation [96]. SIRT6 knockout mice exhibit symptoms of premature aging and succumb to premature death due to severe hypoglycemia [97]. SIRT6 overexpression in male mice has the potential to extend the lifespan by interfering with IGF-1 signaling [98]. By deacetylating the transcription factor GABPβ1, SIRT7 controls the expression of nuclear-encoded mitochondrial genes and provides defense against mitochondrial diseases [99]. Recent studies have highlighted the multifunctional role of sirtuins in IVD metabolism. They coordinate stress responses by collectively regulating substrate clusters rather than by isolating key metabolic enzymes [100]. Moreover, their dependence on NAD^+^ links their enzymatic activity to the metabolic state of the cells, rendering them stress sensors [101]. Consequently, a deeper exploration of the regulation of IVD metabolism is warranted, especially in maintaining mitochondrial homeostasis.

### Acetylation maintains mitochondrial metabolism

**Fig. 4.**
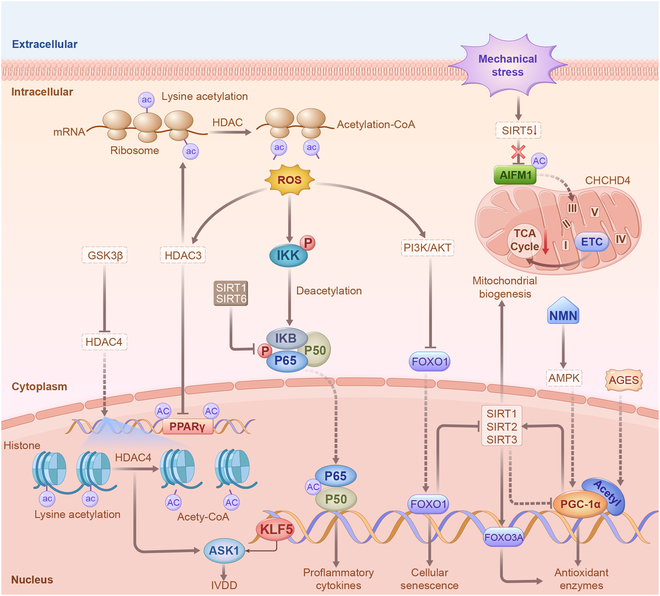
Acetylation regulation of inflammation and maintenance of mitochondrial homeostasis. The deacetylation of lysine in the p65 subunit of NF-κB inhibits its transcriptional activity, mitigating the ensuing inflammatory response. The deacetylation of FOXO3 enhances the expression of FOXO3-dependent antioxidant enzymes and catalase, thereby reducing ROS generation. SIRT1 and SIRT2 elevate the expression of antioxidant enzymes by deacetylating PGC-1α. Mechanical loading increases AIFM1 acetylation in NPCs by suppressing SIRT5 expression, disrupting the interaction between AIFM1 and CHCHD4, and resulting in reduced mitochondrial ETC complex subunits. ROS overproduction induces nuclear translocation of HDAC3, augmenting the association between HDAC3 and PPARγ in NPCs.

Mitochondria play a crucial role in cellular activities by producing ATP through oxidative phosphorylation to ensure normal cellular function [102–103]. However, mitochondrial dysfunction or abnormal electron leakage can lead to oxidative stress, causing damage to cells and triggering cellular senescence [104]. In response to oxidative stress, SIRT2 is up-regulated, leading to deacetylation of FOXO3a and enhanced expression of FOXO3a-targeted genes, ultimately reducing ROS production [105]. As an effector of SIRT2, PGC-1α is indispensable for cell viability and participates in mitochondrial biogenesis and energy regulation [106–107]. SIRT2 protects chondrocytes from apoptosis triggered by oxidative stress by targeting PGC-1α to inhibit mitophagy. By up-regulating antioxidant enzymes such as manganese SOD, SIRT1 deacetylates PGC-1α to suppress oxidative stress [108]. Additionally, SIRT1 removes acetyl groups from FOXO3a, transporting them to the nucleus and enhancing the production of additional antioxidant enzymes and catalase to shield cells against damage induced by oxidative stress [109]. When cellular energy demand increases, the NAD^+^/NADH ratio also increases, leading to heightened SIRT3 activity [110]. Elevated SIRT3 levels during mitochondrial stress enhance the expression of SOD and catalase, reducing ROS buildup during stressful conditions [111]. By targeting AIFM1, SIRT5 disrupts its interaction with CHCHD4, consequently reducing electron transport chain (ETC) complex subunits and impairing mitochondrial function in NPCs, thereby contributing to IVDD progression under mechanical stress [112]. Notably, mechanical stress refers to the physical loading experienced by NPCs within the IVD. NPCs play a pivotal role in maintaining ECM and metabolic homeostasis within the IVD. Upon exposure to mechanical stress, such as repetitive loading or abnormal mechanical forces, aberrant acetylation may serve as a baroreceptor signal, triggering various metabolic and molecular alterations in NPCs, ultimately culminating in IVDD [113]. In addition to sirtuins, acetylation of other proteins, as revealed by proteomic studies, is involved in cellular metabolism. For instance, KLF5 contributes to cartilage degradation by mediating p65 acetylation in the NF-κB cascade activated by IL-1β [114]. Inhibition of HDAC by GSK3β down-regulates KLF5 and ASK1 and prevents IVDD progression [115]. In response to TBHP stimulation, the increased nuclear movement of HDAC3 strengthened its interaction with peroxisome proliferator-activated receptor γ (PPARγ), and HDAC3 knockdown increased PPARγ acetylation and trans-activation, effectively reversing oxidative stress in NPCs (Fig 4). Notably, considering that cofactors such as acyl-CoA donors are metabolites, the cellular metabolic state also plays a vital role in determining the targets and kinetics of acetylation. However, the relevant content of metabolite-mediated PTMs is beyond the scope of this review [116].

## Glycosylation

### Glycosylation regulates ECM metabolism

Protein glycosylation involves the reaction of sugar chain molecules with protein amino acid side chain active groups catalyzed by glycosyltransferase, forming glycosidic bonds that connect sugar chains to proteins. Based on the type of chemical bonds formed during glycosylation, they can be mainly divided into N-glycosylation and O-glycosylation [117]. N-glycosylation occurs on an asparagine residue in the presence of an asparagine-X-Ser/Thr motif (where X is any amino acid other than proline) and is predominantly found in secreted and membrane-bound glycoproteins. O-glycosylation mainly occurs in the Golgi apparatus on serine or threonine residues via the action of glycosyltransferases [118]. This glycosylation pattern is evident in mucosal secretions and transmembrane glycoproteins found on cell surfaces, particularly where polysaccharides are exposed to the external environment. Within the IVD, structural integrity and biomechanical properties rely on two key constituents of the ECM: aggrecan and collagen II [119]. Aggrecan, a proteoglycan, plays a pivotal role in retaining water molecules within the disc, thereby creating osmotic pressure that helps resist stress [36]. Meanwhile, collagen II provides the disc with tensile strength and structural support, enhancing its overall elasticity and capacity to endure mechanical loads [4]. These components synergistically increase the osmotic pressure of the disc, provide essential structural support, and confer elasticity, collectively enabling the disc to effectively function as a load-bearing structure within the spine. However, in IVDD, the accumulation of glycosylation products chemically modifies the aggregate proteins and collagen II, hindering their ability to repair and renew. This imbalance allows ECM breakdown to outpace the building process [120]. Moreover, the up-regulation of catabolic enzymes in the matrix further promotes ECM degradation and accelerates IVDD progression. Studies have reported that the accumulation of glycosylated alterations in aggrecan and collagen II in the IVD increases with age and is partially influenced by sugars. In individuals with diabetes, increased levels of blood sugar expedite the buildup of glycosylation substances [121]. The buildup of glycosylation in NPCs enhances MMP2 expression through the extracellular signal–regulated kinase (ERK) signaling pathway, leading to ECM degradation. In diabetic IVD, MMP13 is up-regulated by BRD4, transmitting MAPK and NF-κB signals and inducing autophagy [122]. Additionally, BRD4 inhibition prevents ECM degradation in diabetic rats. Interestingly, pyridoxamine, as a glycosylation inhibitor [123], effectively alleviates the up-regulation of ADAMTS5 and MMP13, delaying IVDD progression in diabetic mice.

### Glycosylation regulates endoplasmic reticulum stress

**Fig. 5.**
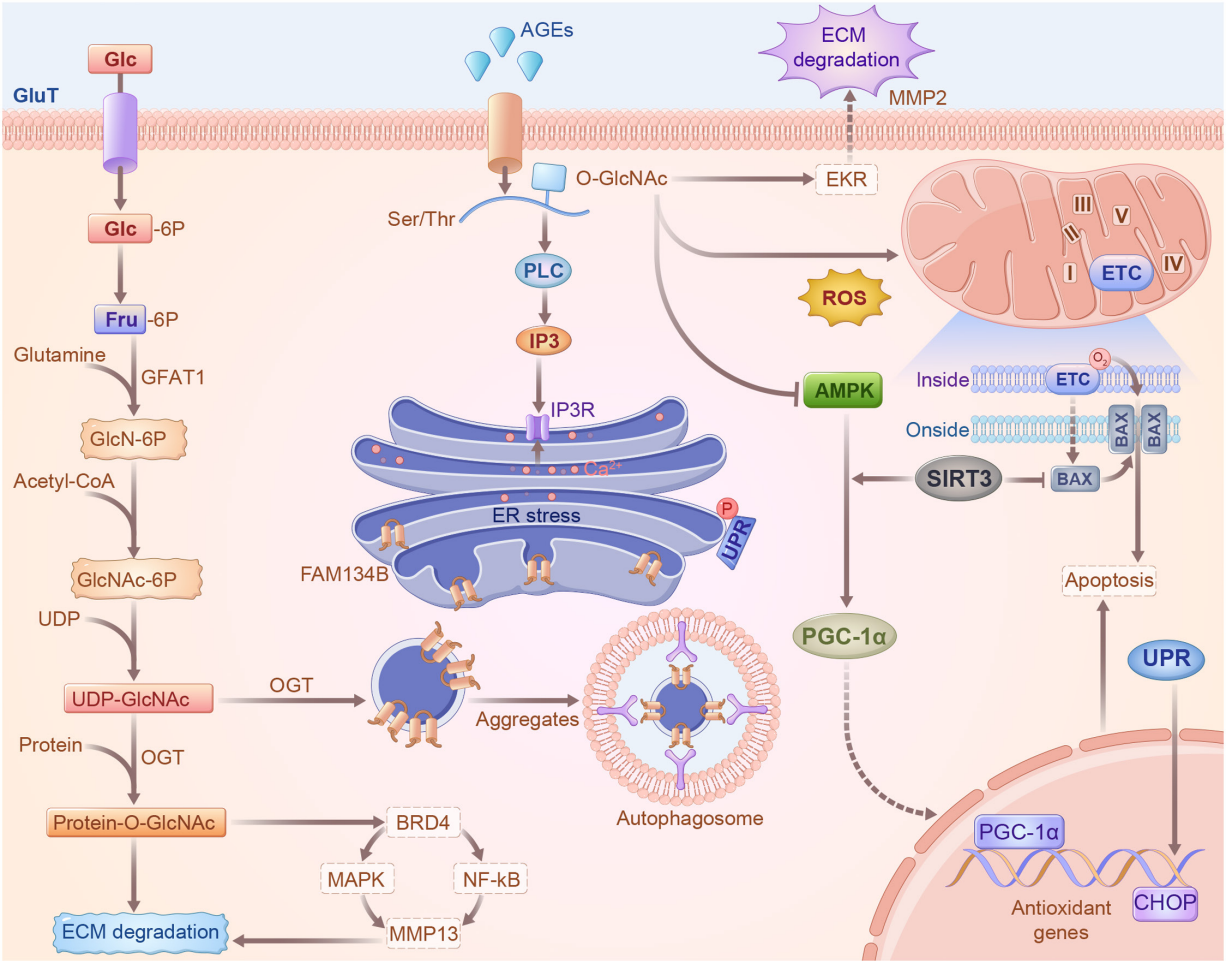
Glycosylation regulation of ECM metabolism and ER stress. In degenerated IVD, glycosylation products predominantly accumulate in aggrecan and collagen II, hindering their repair and renewal. Consequently, the up-regulation of matrix catabolic enzymes occurs, leading to enhanced ECM degradation and accelerated IVDD. Notably, O-GlcNAc glycosylation and O-GlcNAc transferase expression profiles are significantly increased in degenerated NPCs. Furthermore, FAM134B-mediated activation of ER phagocytosis was modulated by O-GlcNAc glycosylation. Following UPR initiation, the downstream C/EBP homologous protein is transcriptionally activated, controlling the expression of apoptosis-related genes and inducing apoptosis under severe ER stress.

The accumulation of glycosylated proteins in IVD tissues affects endoplasmic reticulum (ER) homeostasis, leading to the buildup of unfolded/misfolded proteins, known as ER stress [124]. ER phagocytosis—a form of selective autophagy—involves phagocytosis of specific ER fragments by autophagosomes through specific receptors. These fragments are then transported to lysosomes for degradation, restoring cellular energy levels and ER homeostasis [125]. O-GlcNAc glycosylation, a form of O-glycosylation, and ER-phagy are well-characterized adaptive regulatory mechanisms that help maintain cellular homeostasis and function under various stress conditions. Research has revealed that O-GlcNAc glycosylation and O-GlcNAc transferase expression profiles are significantly increased in degenerated IVD tissues and nutrient-deprived NPCs [126]. Interestingly, increased O-GlcNAc glycosylation abundance can significantly enhance cellular function and promote survival under nutrient-deprived conditions. Additionally, FAM134B-mediated activation of ER phagocytosis is regulated by O-GlcNAc glycosylation, and knockdown of FAM134B, which inhibits ER phagocytosis, significantly counteracts the protective effect of O-GlcNAc glycosylation [127]. As glycosylation products accumulate, ER stress activates the unfolded protein response (UPR) through key transmembrane proteins [128]. After UPR is initiated, the downstream C/EBP homologous protein is transcriptionally activated and controls the expression of apoptosis-related genes, inducing apoptosis under severe ER stress [129–130]. Furthermore, FAM134B overexpression attenuated ROS accumulation, apoptosis, and senescence in NPCs caused by glycosylation products (Fig. 5). However, the current understanding of specific structure–function relationships and the role of specific glycans on target proteins is incomplete. Further global analyses of the enzymatic network coordinating protein glycosylation and assessment of the potential glycosylation energies of specific cell types are required.

## Methylation

### Methylation regulates aging-related gene transcription

**Fig. 6.**
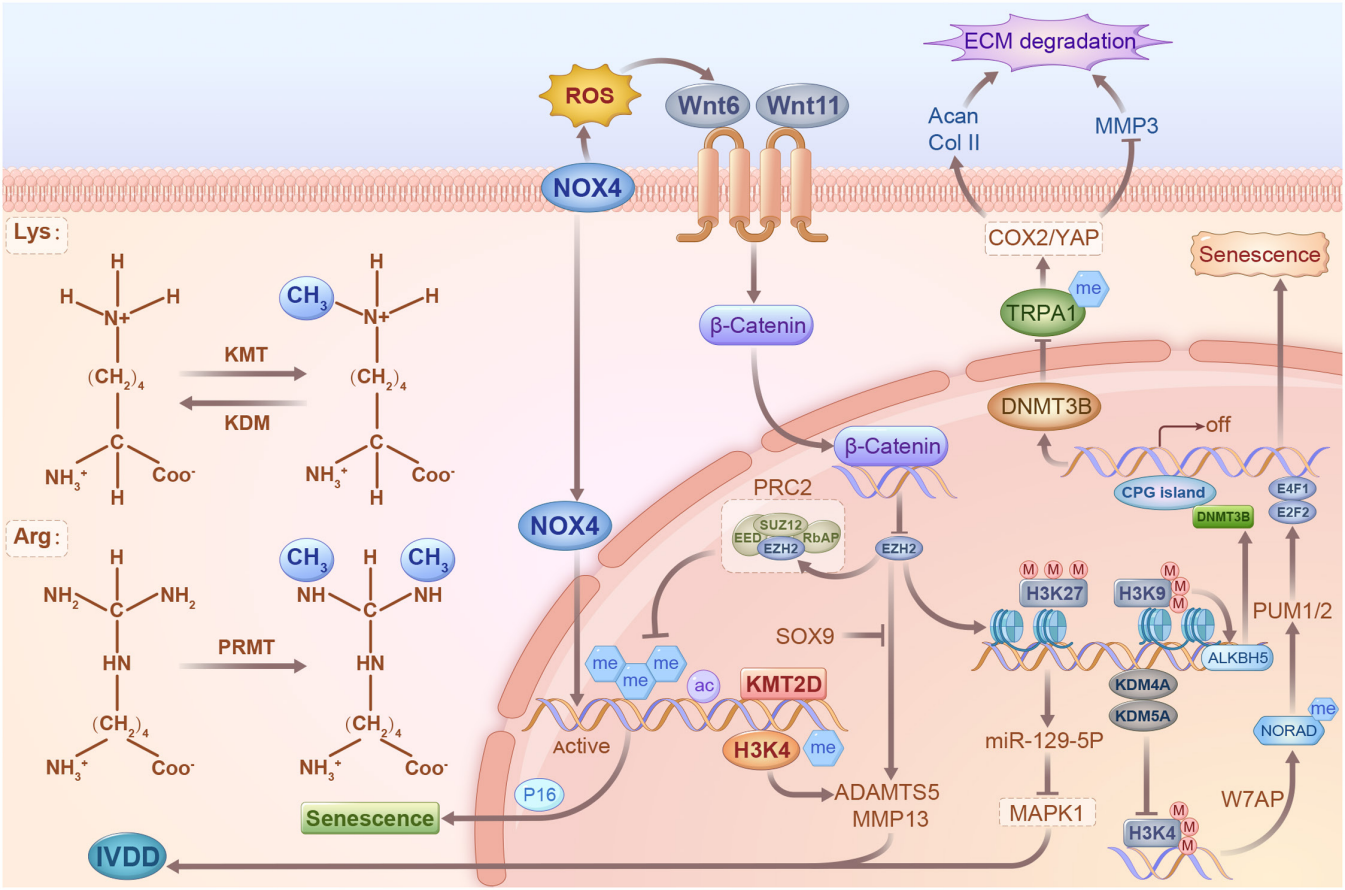
Methylation regulation of age-related metabolism. Methylation undergoes significant alterations in oxidative stress-induced IVDD and is implicated in regulating the expression levels of catabolic enzymes such as MMP3, MMP9, and MMP13. The feedback loop between EZH2 and NOX4 regulates NPC senescence. Additionally, EZH2 overexpression inhibits the expressions of collagen II and aggrecan while promoting the expressions of ADAMTS5 and MMP13. SOX9 overexpression reversed the effects of EZH2 in rat NPCs. Moreover, miR-129-5p overexpression or MAPK1 silencing promotes NPC proliferation and inhibits senescence. Intriguingly, miR-129-5p down-regulates MAPK1 expression, whereas EZH2 inhibits miR-129-5p by modifying H3K27me3 in the promoter, increasing the release of inflammatory and cellular senescence factors.

Protein methylation is a common covalent PTM catalyzed by methyltransferases that add methyl groups from the donor S-adenyl-L-methionine to specific substrates [131]. These enzymes can be divided into lysine methyltransferases (KMTs) and arginine methyltransferases (PRMTs) based on their target residues [132–133]. Abnormal methylation levels often occur in aging cells, and their up-regulation is associated with a poor prognosis [134]. In the context of IVDD, several studies have highlighted the role of protein methylation in regulating cellular processes. For instance, WTAP expression increases in aging NPCs due to a KDM5A-mediated epigenetic increase in promoter H3K4me3, contributing to NORAD posttranscriptional regulation [135]. Another study revealed that the ALKBH5 expression is enhanced during IVDD due to decreased KDM4A-mediated H3K9me3 modification. Functionally, ALKBH5 induces NPC senescence by demethylating DNMT3B transcripts, promoting its expression via reduced YTHDF2 recognition and subsequent degradation due to transcript hypomethylation in vitro and in vivo [136]. DNMT3B, which inhibits COX2 expression by methylating the TRPA1 promoter, is suggested to promote NPC proliferation through the TRPA1/COX2/YAP axis, potentially preventing ECM degradation and alleviating IVDD in rats [137]. KMT2D and H3K4me1 modifications were significantly up-regulated during oxidative stress-induced degeneration of NPCs, and knockdown of KMT2D down-regulated the expression levels of catabolic enzymes, such as MMP3, MMP9, and MMP13 [138], highlighting the crucial role of KMT2D-mediated methylation in the pathological state of IVD [139]. EZH2, which regulates NOX4 expression through H3K27me3 at the promoter, is involved in a feedback loop with NOX4, which regulates NPC senescence [140]. EZH2 overexpression inhibits the expressions of collagen II and aggrecan, thereby promoting the expressions of ADAMTS5 and MMP13. However, SOX9 overexpression can reverse the effect of EZH2 in rat NPCs [141]. Additionally, miR-129-5p expression is reduced, while EZH2 and MAPK1 levels are overexpressed in the lumbar spine tissue of patients with IVDD [142]. Mechanistically, miR-129-5p can down-regulate MAPK1 expression, whereas EZH2 inhibits miR-129-5p by modifying H3K27me3 in the promoter, increasing the release of inflammation and cell senescence factors (Fig 6). Methylation regulation in IVDD primarily focuses on histones, which are closely associated with chromatin remodeling. Histone methylation, particularly at the N-terminal tails of H3 and H4, modifies and remodels chromatin by recruiting specific reading proteins or enzymes, thereby regulating chromatin accessibility and gene transcription. However, an increasing number of studies have reported methylation events in nonhistone proteins, revealing that methylation is involved in various biological processes.

**Fig. 7.**
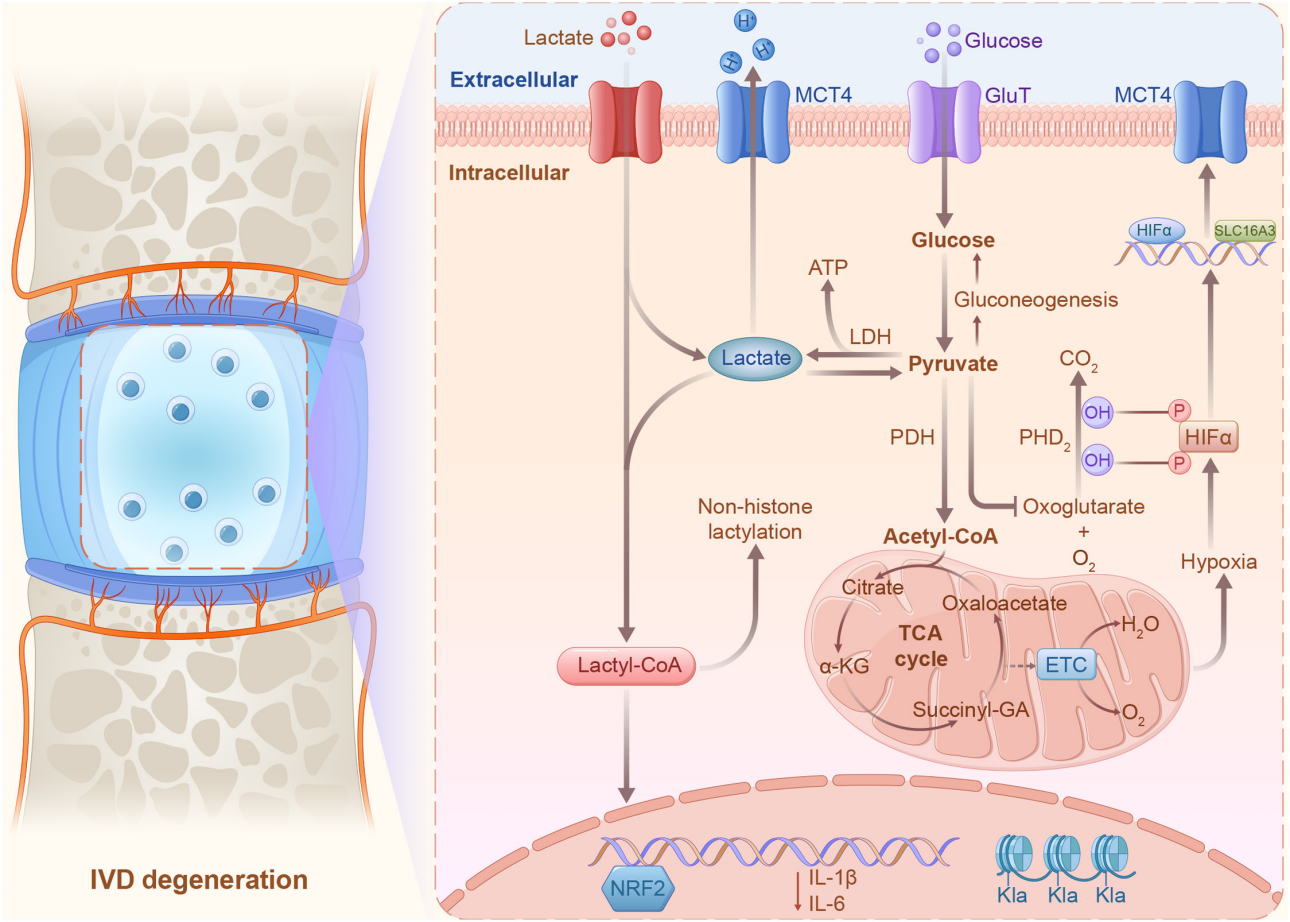
Hypoxia-induced lactate production regulating anti-inflammatory repair. Glucose is taken up by NPCs by the glucose transporter (GluT), and glycolysis converts glucose to pyruvate. Lactic dehydrogenase (LDH) converts pyruvate to lactate, which is exported from NPCs by MCT4. Lactate can be converted to lactate-CoA, which participates in the lactylation of histone and nonhistone proteins. This process regulates the expression of genes encoding anti-inflammatory factors such as NRF2. Conversely, LDH1 converts lactate to pyruvate, which is then converted to acetyl-CoA via pyruvate dehydrogenase (PDH). Pyruvate dehydrogenase kinase (PDK) negatively regulates the entry of acetyl-CoA into the TCA cycle, generating precursors for biosynthesis and/or oxidative phosphorylation for ATP production.

## Lactylation

### Lactate regulates energy metabolism

Given the avascular nature of IVD, energy metabolism in this tissue primarily relies on glycolysis for survival, leading to a high production of lactate [143]. Lactate is traditionally regarded as a terminal waste product, contributing to lowering pH value within the IVD. This acidic environment inhibits proteoglycan synthesis, increases MMP activation, promotes cellular senescence, and ultimately accelerates IVDD [2,14–15]. Conventionally, in a nutrient-poor and hypoxic setting, heightened cellular demand results in increased accumulation of lactate, which serves as the end product of anaerobic glycolysis. However, recent studies have revealed contrasting responses in healthy IVD. Following exercise, lactate levels exhibited an immediate decrease, slowly and partially normalizing for 1 hour. This intriguing finding suggests that lactate, instead of being regarded solely as a waste product necessitating excretion, may be utilized as a fuel source through alternative pathways [144]. Alterations in this response hold promise as potential noninvasive radiological biomarkers for the early detection of disc degeneration. Moreover, the burgeoning interest in treatments targeting IVD degeneration and regeneration has spurred a deeper exploration of the internal metabolic mechanisms within the disc environment at the molecular level. Elevated lactate levels act as a hypoxia mimetic factor, promoting the biosynthesis of tricarboxylic acid (TCA) cycle intermediates that functionally compete with α-ketoglutarate, which is required for HIF-1α hydroxylation and degradation. During the final steps of glycolysis, HIF-1α controls the flow of lactate and H^+^ out of NPCs, partially regulating glycolytic flux [145]. HIF-1α increases SLC16A3 expression, which encodes monocarboxylate transporter 4 (MCT4) that couples H^+^ and lactate. However, rapid suppression of MCT4 results in decreased glycolysis and TCA cycle flow, causing reconfiguration of metabolic processes in NPCs. NPCs exhibit metabolic plasticity, with short-term MCT inhibition leading to up-regulated TCA cycle flux and maintained oxidation–reduction reaction ratios. However, prolonged MCT4 inhibition may impair NPC viability due to cytoplasmic acidification and failure to maintain a high TCA cycle flux. Elimination of MCT4 in mice reproduces the key pathoanatomical characteristics of IVDD in humans, such as the absence of cell phenotypic markers and disruption of ECM integrity [146–147]. Studies on NPCs have revealed that the interplay between metabolic flow and HIF-1α function is regulated by cytosolic lactate levels [148]. Intracellular lactate accumulation enhances HIF-1α function, whereas the activation of MCT4 transcription is controlled by intronic enhancers sensitive to HIF-1α. This suggests a reciprocal relationship between MCT4 activity induced by hypoxia and the stability of HIF-1α in NPCs, with inherent lactate levels controlling this relationship. Therefore, cytosolic lactate levels play a crucial role in modulating the interplay between metabolic flow and HIF-1α function in NPCs.

### Lactylation regulates inflammatory microenvironment

Recent scientific discoveries have unveiled how lactate-derived histone lactylation serves as a mediator of gene expression regulation. Lactylation disrupts histone–DNA interactions and activates transcriptional machinery under specific conditions [149]. In NPCs, blocking glycolysis leads to reduced lactate production in the IVD microenvironment, potentially influencing IL-1β production by macrophages. Several studies have demonstrated the significant impact of macrophage-mediated inflammation on NPC function and viability in IVD. For example, macrophage polarization regulates IVDD by modulating cell proliferation, inflammatory mediator secretion, and the ECM [150]. Macrophage infiltration into degenerative IVD is correlated with the severity of disc degeneration and is associated with increased inflammatory cytokine expression and ECM degradation [151]. These studies provide evidence of the indirect effects of macrophages on NPCs through the secretion of proinflammatory mediators and modulation of the disc microenvironment. However, histone lactylation in macrophages, especially in classically activated proinflammatory macrophages, enhances the expression of genes related to wound healing, such as ARG1 [152–153]. Research indicates that proinflammatory macrophages transition to a homeostatic selective activation state during later stages, contributing to inflammation resolution [154]. This aligns with previous findings linking lactate to the polarization of specific macrophages [155]. The absence of direct blood supply in the IVD creates a distinctive microenvironment marked by inadequate oxygen levels and hindered nutrient diffusion [122]. Consequently, cells residing within the IVD, including resident macrophages, predominantly resort to glycolysis as the primary means of energy production under hypoxic conditions, leading to lactate generation as a metabolic byproduct. Lactate accumulation within the compact and oxygen-deprived milieu of IVD has the potential to reshape the epigenetic landscape of resident macrophages. Initially, proinflammatory gene expression relied heavily on glycolysis. However, as time progresses, lactate emerges as a key regulator, with its abundance—stemming from glycolysis—exerting an anti-inflammatory effect through the lactylation of histones at specific target genes [156]. This lactylation-mediated anti-inflammatory and pro-repair response of NPCs warrants further investigation. Moreover, considering the altered TFEB-dependent autophagy observed in IVDD and the regulatory role of lactate in autophagy, it becomes essential to investigate the direct or indirect role of cellular lactate in IVDD [157]. The intricate interplay between lactate, histone lactylation, and inflammation resolution holds potential significance for understanding and addressing IVDD.

## Conclusions and Prospects

As previously mentioned, PTMs have been associated with IVDD, outlining their regulatory roles in NPCs and chondrocytes. These PTMs intricately contribute to the regulation of protein function and ensure precise functionality at designated times and locations. Consequently, they introduced novel and diverse mechanisms crucial for sustaining metabolic homeostasis within the IVD. By delving into the mechanisms of PTMs governing metabolism and inflammatory responses in NPCs and chondrocytes, this discussion sheds light on the intricate ways in which PTM-driven alterations in metabolism and inflammation impact IVDD. For instance, by altering the charge state of a protein, phosphorylation influences its interactions with other molecules, thereby regulating key enzyme activities in metabolic pathways. Within the intricate metabolic signaling network, the extensive interplay of key kinases emerges as a prominent characteristic of phosphorylation in regulating IVD metabolism. As an inflammatory regulator, ubiquitin can be conjugated to target proteins to mark them for proteasomal degradation. This process directly influences intracellular protein levels, thereby regulating metabolic status and cell survival mechanisms to counter the impact of oxidative stress and inflammation on the IVD. Abnormal glycosylation levels during IVDD lead to an excessive folding burden on the ER, which is responsible for the correct folding of newly synthesized proteins into functional structures. Sirtuin-mediated acetylation plays a multifunctional regulatory role in metabolism, encompassing energy balance, lipid and glucose metabolism, and resistance to oxidative stress. These molecules have emerged as key regulators of cell metabolism and are essential for maintaining normal metabolism in IVD, particularly mitochondrial homeostasis. The regulatory impact of methylation in IVDD primarily focuses on histones, which induce chromatin to form a condensed structure that limits DNA accessibility, thereby playing a pivotal role in biological processes such as the cell cycle. Lactate, the main product of glycolysis, mediates lactylation in metabolic processes and exhibits anti-inflammatory and repair-promoting effects. Some researchers have highlighted the anti-inflammatory and antioxidative properties of lactate in mitigating disc degeneration and promoting tissue repair in preclinical IVDD models. Strategies aimed at modulating lactate levels, such as the inhibition of lactate dehydrogenase or augmentation of lactate utilization pathways, have demonstrated promise in preclinical studies for attenuating disc degeneration and promoting tissue regeneration. Given the limited number of existing studies, the role of lactylation in IVDD is a key area warranting further research. In summary, these modifications exert regulatory control over IVD metabolism by altering the structure, stability, or interactions of proteins. The specific nature and mechanism of each modification contribute to these differences.

Notably, the interaction of different PTMs leads to joint regulatory effects, contributing to a more intricate and sophisticated regulation of cellular metabolism. The significance of this joint regulation lies in the integration of multiple signaling pathways, ensuring cellular adaptability to complex environmental changes. PTMs exert control over proteins involved in cell signaling either by activating or inhibiting specific signaling pathways. This synergistic effect directly influences the cellular responses to external stimuli, thereby regulating energy metabolism and cell growth. Furthermore, PTMs may occur at specific cellular locations or during particular life cycle stages, achieving spatiotemporal regulation of metabolism. This regulatory approach enhances cellular sensitivity to environmental changes, enabling cells to effectively respond to diverse environmental stressors and physiological requirements. A comprehensive understanding of these PTM interactions not only unravels the complexity of cellular regulatory networks but also promises novel insights for future biomedical research and IVDD treatment. Additionally, metabolites or intermediates generated through glycolysis, the TCA cycle, lipid and amino acid metabolism, and other pathways act as signaling molecules, impacting the PTMs of proteins. Although it is beyond the scope of this review, elucidating these processes holds the potential to deepen our understanding of IVDD’s nature, offering profound considerations for future treatments and management strategies. The knowledge gained from current metabolism-related studies suggests that PTMs may be more prevalent and crucial to cell biology than initially perceived. We posit that given the appropriate substrates and reaction conditions, PTMs occur in all biomolecules, irrespective of their intracellular location. In addition to enzyme availability, substrate availability may dictate the type and extent of PTMs. Consequently, the pursuit of new PTMs should be guided more by substrate availability rather than by enzymes. Future studies should leverage multi-omics mapping sequencing with high spatial resolution, proteomics, and metabolomics for the in-depth phenotypic analysis of IVDD. In this regard, innovative technologies are imperative to capture these PTMs, such as the development of biorthogonal labeling techniques coupled with microscopy imaging or high-resolution mass spectrometry. Establishing frameworks for the detection, treatment, and prevention of challenging human diseases is pivotal in this evolving landscape.
